# Design, Characterization and Applications of Functional Nanomaterials

**DOI:** 10.3390/molecules26237097

**Published:** 2021-11-24

**Authors:** Marco Fabbiani, Federico Cesano, Francesco Pellegrino, Chiara Negri

**Affiliations:** 1 ICGM, CNRS, ENSCM, University of Montpellier, 34095 Montpellier, France; marco.fabbiani@umontpellier.fr; 2 Department of Chemistry, Università di Torino, Via Pietro Giuria 7, 10125 Torino, Italy; federico.cesano@unito.it; 3 Department of Energy, Politecnico di Milano, Via La Masa 34, 20156 Milano, Italy; chiara.negri@polimi.it

**Keywords:** nanomaterials, catalysis, photocatalysis, electrocatalysis, energy, food, medicine, hybrid materials, computer-aided molecular design of nanomaterials, machine learning for material synthesis and application

Nanomaterials are commonly defined as particles existing in nature or artificially manufactured materials that have one or more external dimensions in the 1–100 nm range [1,2]. Owing to their size constraint, nanostructures exhibit a high specific surface area combined with new optical, electronic, magnetic, mechanical properties, and catalytic activity as compared with their 3D counterparts. All these emerging properties have the potential to revolutionize many fields [2]. In this regard, the research on nanomaterials broadly covers a wide range of science, exploiting several sectors from medicine to materials science and technology. Nanosized structures need to be investigated to understand their potential impact on the environment and human health as well as their safe use and handling [2,3]. A vast amount of research has also been dedicated to investigating the properties and applications of such new nanomaterials. In December of 1959, Richard Feynman prospected the handling down to the single-atom scale. This date has been commonly recognized as the “birth” of nanotechnology; however, more than two decades elapsed before the first recognized paper on molecular manipulation was published. Since then, nanomaterials and nanotechnology have received increasing interest, and, today, their applications in automotives, aerospace, biomedicine, (bio)sensors, computer chips, energy harvesting and conversion, medical implants, and smart textiles are feasible [4,5,6,7].

The present Special Issue contains three reviews and five research articles emphasizing achievements and future developments in the field of the design, characterization, and applications of nanomaterials. 

Regarding the energy applications of nanoparticles, this Special Issue contains some contributions. Fornasiero et al. [8] highlighted the existence of various classes of nanostructured gels and the most recent advancements in the field. In particular, the authors focused on the future directions of this challenging area. Nanostructured gels are emerging as attractive, functional materials that could have several uses in the field of energy, with applications ranging from extraction and purification to catalysis. The nanogel characteristics can be tuned by changing different aspects, such as the gelator types and concentrations, pH, temperature, gelling methods, etc. For example, ad hoc modifications can be considered to create flexible materials suitable for coating innovative and stretchable electronic devices. Nanostructured gels could also be employed to study the interconnection among energy sources, sustainability, and pollution. Indeed, many efforts have been made to explore new, greener methods and bypass the strong energy dependence on fossil fuels. Hydrogels, mainly made of water, represent a great opportunity for the development of clean energy systems. In this context, gels can act as innovative materials for energy and water technologies, offering their 3D networks as a stable support for promoting ion and electron transfers in energy devices and as a modern tool in water purification.

Other contributions in the energy field come from papers by Rorabeck et al. [9] and Santalucia et al. [10]. The first research paper showed the fabrication of nanostructured composite electrodes based on Mn_3_O_4_ and multiwalled carbon nanotubes (MWCNTs) by using the salting-out method, which makes the particle transfer from an aqueous synthesis medium to a 2-propanol suspension possible. Octanohydroxamic acid (OHA) was used as both a capping and dispersing agent for the synthesis and particle extraction steps. According to the adopted method, the adsorption of OHA on Mn_3_O_4_ particles involved the formation of Mn complexes on the surface, thus facilitating the particle transfer into the 2-propanol phase. The authors observed an increase in capacitance during the cycling of the fabricated electrodes together with good capacitance retention at high scan rates and cyclic stability. Santalucia et al. [10], in their paper, investigated the in-situ photoreduction of Mo^6+^/SiO_2_ in the presence of CO by means of FT-IR and UV-vis diffuse reflectance spectroscopies. Combining these complementary techniques, they were able to monitor the changes of Mo oxidation states and coordination over time. The results showed that UV-vis irradiation promotes the formation of a Mo^5+^ state which—interacting with CO—can form CO_2_. These changes between Mo^6+^/Mo^5+^ were proven by the disappearance of the ligand–to–metal charge transfer features of isolated Mo^6+^/SiO_2_. Furthermore, by simultaneously collecting FT-IR spectra and irradiating with a UV-vis beam, the authors were able to monitor the subsequent reaction step, proving the conversion of the formed Mo^4+^(CO)_3_ complexes to Mo^4+^(CO)_2_. These results significantly contribute to the understanding of Mo^6+^ redox properties, which can be exploited in various (photo)catalytic processes.

The last contribution in the energy field comes from Barreca et al. [11]. In their work, the authors tackled the mechanism of metal-ligand bond dissociation in a Zn complex with β-diketonate and diamine ligands. Such systems find their application in the chemical vapor deposition (CVD) processes for the preparation of ZnO nanosystems. By means of density functional theory (DFT) and ab initio molecular dynamics (AIMD) coupled with a statistical sampling approach, they explored the potential energy surface of the octahedral–to–square pyramidal conversion on a hydroxylated silica slab. The simulations carried out at 500 K showed an interconversion barrier of the same order of magnitude of the thermal energy at the operating temperature, where the hydrogen bond of the support surface plays a key role in the dissociation of the Zn–O bond. The atomistic insights provided in this contribution could be of crucial importance for tailoring the design of the first nucleation stages of the targeted Zn–O nanostructures.

Jing and Shi contributed to this Special Issue with a research paper concerning metal oxide nanoparticles for environmental applications [12]. The authors prepared functionalized Fe_3_O_4_ nanoparticles to achieve a classified and easy recovery of heavy metal ions from wastewater. They proposed a facile approach to graft Tris [2-(dimethylamino) ethyl] amine (Me_6_TREN) onto the surface of SiO_2_-coated Fe_3_O_4_ nanoparticles, which were subsequently employed as nano-adsorbents. The coating of Me_6_TREN is responsible for the immobilization of heavy metal ions at the surfaces of nanoparticles, and the absorption efficiency is high and with good selectivity, while the Fe_3_O_4_ core allowed for the magnetic manipulation of the material. Furthermore, Fe_3_O_4_@Me_6_TREN NPs can be regenerated by desorbing heavy metal ions from NPs with EDTA sodium salt, which will be of great significance for cost reduction and further industrial applications.

Another application field of nanoparticles is in the biomedical domain. A biomedical application of nanoparticles was reported by Day et al. [13]. They studied the design and synthesis of novel mesoporous silica nanoparticles charged with the hormonal drug tamoxifen in order to facilitate the guidance towards estrogen receptors (ER), which are upregulated in breast tumors. The authors reported the conceptual design, synthesis, and preliminary physical and chemical characterization of a novel nanostructure that could serve as a platform for future drug delivery systems (DDSs). Its uniqueness derives from the combination of successful synthesis methodology and targeting strategies. This study demonstrates a new theoretical concept for a DDS, based on: (i) passive targeting using nanosized silica nanoparticles as carriers, (ii) active targeting employing tamoxifen as a targeting vector to selectively bind to ER-overexpressing breast tumors, and (iii) pH-controlled drug release by grafting poly(L-histidine) onto the surface of silica to act as a pH-sensitive gatekeeper. The authors concluded that the triple-action system holds remarkable promise as a drug delivery vector for new cancer therapies.

Always inherent in the biomedical field, the review proposed by Chircov et al. [14] is focused on mesoporous silica nanoparticles (MSNs). Indeed, their tunable porosity, as well as the possibility for surface functionalization and the pore nature to allow for the encapsulation of bioactive molecules, make MSNs ideal candidates for drug delivery systems. A step forward analyzed in the review is the case of stimuli-responsive systems for selective delivery through the addition of specific triggering factors in the case of cancer therapy treatments. The authors also addressed the need to develop materials for antibiotic removal from wastewaters, and therefore, the use of MSNs in environmental applications, such as the removal and degradation of hazardous agents (e.g., antibiotics and pesticides).

The third application field of nanoparticles highlighted in this special issue is food manufacturing. Pateiro et al. [15] reviewed nanoencapsulation-based technologies applied to food products. Nanoencapsulation, which can be defined as the confinement of a bioactive compound (BAC) in a matrix, can have a wide range of applications in the food sector, from preserving food safety to improving organoleptic properties. The authors reported the (i) applications, (ii) classification, (iii) utilized coating, and (iv) bioactive compounds of nanoencapsulation systems and techniques. While discussing the great advantages, the authors also focused on the existing challenges—from the elevated costs and complexity of the systems to the still limited data on food safety, risk assessment, and toxicological and ethical issues—pointing to the benefit that society would experience from the application of nanomaterials in food production when covering these gaps.

As we have highlighted, nanoparticles have an ever greater impact in various fields of science and technology. In this Special Issue, only some nanoparticle topics have been covered. However, we believe that the study and use of nanoparticles will be increasingly widespread in the context of industrial and non-industrial processes, especially from the perspective of climate change mitigation.
